# Hybrid nanostructured particles via surfactant-free double miniemulsion polymerization

**DOI:** 10.1038/s41467-018-04320-7

**Published:** 2018-05-15

**Authors:** Yongliang Zhao, Junli Liu, Zhi Chen, Xiaomin Zhu, Martin Möller

**Affiliations:** DWI – Leibniz-Institute for Interactive Materials e.V., Institute for Technical and Macromolecular Chemistry of RWTH Aachen University, Forckenbeckstrasse 50, 52056 Aachen, Germany

## Abstract

Double emulsions are complex fluid systems, in which droplets of a dispersed liquid phase contain even smaller dispersed liquid droplets. Particularly, water-in-oil-in-water double emulsions provide significant advantages over simple oil-in-water emulsions for microencapsulation, such as carrier of both aqueous and oily payloads and sustained release profile. However, double emulsions are thermodynamically unstable systems consisting typically of relatively large droplets. Here we show that nanoscale water-in-oil-in-water double emulsions can be prepared by adding a silica precursor polymer, hyperbranched polyethoxysiloxane, to the oil phase without any additional surfactants. The resulting double miniemulsions are transformed to robust water@SiO_2_@polymer@SiO_2_ nanocapsules via conversion of the precursor to silica and polymerization of the oil phase. Other intriguing nanostructures like nanorattles and Janus-like nanomushrooms can also be obtained by changing preparation conditions. This simple surfactant-free double miniemulsion polymerization technique opens a promising avenue for mass production of various complex hybrid nanostructures that are amenable to numerous applications.

## Abstract (web summary)

Double emulsions show significant advantages for microencapsulation but are thermodynamically unstable. Here the authors show, that silica nanocapsules with nanorattles or Janus-like nanomushroom structures can be prepared by stabilizing double emulsions with a silica precursor polymer and subsequent polymerization of the oil phase.

## Introduction

Double emulsions (also referred to as multiple emulsions) are complex fluid systems, in which droplets of a dispersed liquid phase contain even smaller dispersed liquid droplets^1–3^. Particularly, water-in-oil-in-water (W/O/W)-type double emulsions where each dispersed water droplet is separated from the continuous aqueous phase by a layer of an oil phase can provide significant advantages over simple oil-in-water (O/W) emulsions for microencapsulation applications, such as carrier of both aqueous and oily payloads and sustained release profile^4–8^. Double emulsions are thermodynamically unstable systems consisting typically of relatively large droplets. They are usually prepared using a two-step emulsification process involving the formation of a primary water-in-oil (W/O) emulsion under high-shear conditions and the subsequent dispersion of this W/O emulsion into water with a relatively gentle shear force to avoid rupture of the internal water droplets, while a one-step procedure often leads to badly reproducible results^3^. As each emulsification step results in a highly polydisperse droplet distribution, double emulsions are generally very polydisperse and poorly controlled in structure. Monodisperse double emulsions can be obtained using a microcapillary device with yet compromised productivity^9^. In a typical double emulsion, two sets of surfactants with different hydrophilic-lipophilic balance (HLB) values are used; the hydrophobic surfactant with a lower HLB value is designed to stabilize the W/O interface and the hydrophilic one for the O/W interface. However, these surfactants may interfere with each other, thus affecting the emulsion stability^10^. Quite a few approaches were considered to improve the stability of double emulsions, for example the use of polymeric amphiphiles^11–15^ and interfacial-active solid particles^16–19^ as emulsifiers. It was reported that double emulsions could be obtained by employing single polymeric emulsifiers^12, 13^, possibly because of the distribution of HLB values typical for polymer samples. Nanoscale double emulsions that were stable for months were prepared using amphiphilic diblock copolypeptides, in which the control of hydrogen bond presentation in polypeptide segments acts as a stabilizing factor, though the reduction of droplet size succeeded by passage six times through a microfluidic homogenizer^12^. In the case of W/O/W double emulsions, the oil phase, which is a solution of an organic polymer, can be solidified by solvent evaporation, thus polymer nanocapsules containing water-soluble substances are obtained^17, 20^. Suspension polymerization in the double emulsions was also carried out to yield microsized capsules^21, 22^. However, the formation of robust nanocapsules from the double emulsions has never been demonstrated either by solvent evaporation or by oil phase polymerization techniques.

Recently, we reported the formation of O/W miniemulsions by emulsifying a solution of a silica precursor polymer, hyperbranched polyethoxysiloxane (PEOS)^23^, in a hydrophobic liquid in water^24, 25^. PEOS is a water-insoluble liquid, which becomes amphiphilic upon hydrolysis at the oil/water interface. The interfacial tension between a styrene solution containing PEOS and water of pH 7 reaches with time the final value as low as 10 mN/m that is only 1/3 of the value between pure styrene and water. Monodisperse polystyrene@SiO_2_ core-shell nanoparticles were prepared via miniemulsion polymerization of the styrene/PEOS-in-water system^25^. Since the amphiphilicity of PEOS is induced by hydrolysis at the oil/water interface, PEOS should in principle also be able to stabilize W/O emulsions. In a W/O pickering emulsion stabilized by SiO_2_-nanoparticles, PEOS dissolved in the oil phase migrates to the interface and all-silica colloidosomes enclosing an aqueous phase were thus obtained^26^.

In the present work, PEOS is used to stabilize both internal and external oil-water interfaces in a W/O/W double emulsion. A nanocapsular structure is eventually obtained by polymerization of the oil phase and conversion of PEOS, if a nanoscale double miniemulsion forms. Here the influence of the reaction conditions, such as emulsion composition, shear force, pH of aqueous phases, and so on, on the morphology of the resulting particles is demonstrated. Other intriguing nanostructures like nanorattles and Janus-like nanomushrooms can also be prepared under certain conditions.

## Results

### Preparation of water@SiO_2_@polymer@SiO_2_ nanocapsules

**Fig. 1:**
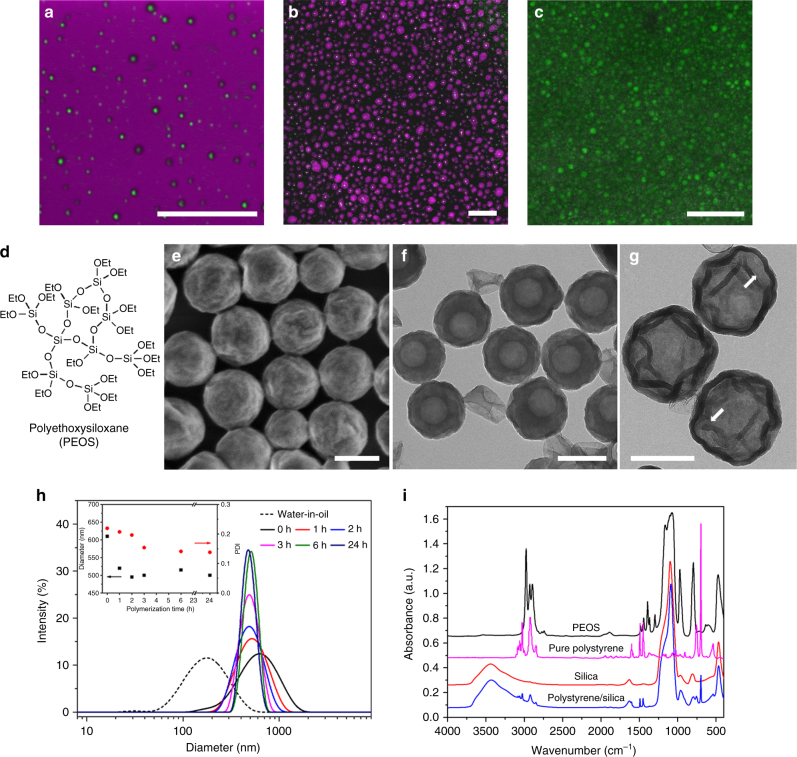
Transformation of a double miniemulsion to nanocapsules. **a**–**c** Fluorescence micrographs of primary water-in-styrene/PEOS emulsion, water-in-styrene/PEOS-in-water double emulsion, and resulting water@SiO_2_@polystyrene@SiO_2_ nanocapsules, respectively. The mass ratio of PEOS to styrene is 1:1, the inner water phase to the oil phase is 1:10, and the primary W/O emulsion to the outer water phase is 1:10. The pH value of the water phase is 7. Pink color represents the oil phase labeled with Nile red and green color shows the aqueous phase labeled with fluorescein sodium salt. Scale bars = 5 μm. **d** Chemical structure of polyethoxysiloxane (PEOS). **e** Field-emission scanning electron microscopy (FESEM) and **f** transmission electron microscopy (TEM) images of the water@SiO_2_@polystyrene@SiO_2_ nanocapsules. **g** TEM image of the particles shown in **e** and **f** after calcination in a muffle oven, where thin silica layers are pointed out by white arrows. Scale bars in the electron microscopy images are 200 nm. **h** Size distribution of the primary W/O emulsion and size evolution of the W/O/W double emulsion during polymerization measured by dynamic light scattering. PDI is polydispersity index. **i** Fourier-transform infrared spectra of PEOS, pure polystyrene, dried water@SiO_2_@polystyrene@SiO_2_ nanocapsules, and silica material obtained after calcination of the nanocapsules at 600 °C in air

Water can easily be emulsified in the solution of PEOS in styrene using ultrasonication. The formed W/O emulsion contains very small water droplets with the median diameter close to 170 nm as shown in Fig. 1a, h. It indicates that PEOS macromolecules can also adapt themselves to the W/O curvature due to limited access to water by hydrolyzing only a minor fraction of hydrophobic ethoxysilane to hydrophilic silanol groups. The catastrophic phase inversion from W/O to O/W type of emulsion takes place if the water fraction is raised above 67%. At lower water fraction, the resulting W/O emulsion is stable against coalescence, and it remains liquid for a couple of hours at room temperature. Due to further hydrolysis and condensation of PEOS, the viscosity of the emulsion increases with time and eventually gelation occurs. The primary W/O emulsion is further emulsified in water before gelation, and another stable emulsion is formed. It can be seen that the internal water droplets labeled with a green fluorescence dye are successfully encapsulated within the pink-labeled oil droplets that are in turn dispersed in a non-labeled external aqueous phase, thus the formation of a stable W/O/W double emulsion (Fig. 1b) is confirmed. In this complex emulsion system, both W/O and O/W interfaces are stabilized by partially hydrolyzed PEOS macromolecules. From the fluorescence optical micrograph (Fig. 1b), the mean diameter of the oil droplets in the double emulsion is determined to be 700 nm, very close to the size measured by dynamic light scattering (DLS) (Fig. 1h). Remarkably, almost each oil droplet encloses only one water droplet most probably because of the comparable size of the internal water droplets and outer oil droplets.

A radical initiator, 2,2′-azobis(2-methylpropionitrile) (AIBN), is dissolved in the oil phase, and the W/O/W double emulsion is heated to 70 °C to induce the radical polymerization of styrene. Meanwhile, the conversion of PEOS at both W/O and O/W interfaces is accelerated. After stirring at this temperature for 24 h, a stable milky dispersion is obtained. Fourier-transform infrared (FT-IR) spectroscopy confirms the full conversion of PEOS to silica and the formation of polystyrene (Fig. 1i). According to electron microscopy data, the nanoparticles formed in the dispersion have an average diameter of 250 ± 22 nm by measuring 1000 particles (data shown is mean ± s.d.) (Fig. 1e, f). Transmission electron microscopy (TEM) shows that they exhibit a well-defined capsular structure that consists of a hollow core of about 110 nm in diameter and a shell with a thickness of ~70 nm (Fig. 1f). After calcination in a muffle oven, hollow silica spheres with a shell thickness of ca. 20 nm are obtained (Fig. 1g), and the overall particle size remains almost unchanged. Thus, the thickness of the organic polystyrene layer, which is removed by calcination, can be estimated to be ca. 50 nm. Furthermore, a very thin silica layer as pointed out by a white arrow in Fig. 1g is observed inside a silica hollow particle. This silica layer is thought to be located around the internal water droplets in the nanocapsules. The resulting microcapsules prepared with an internal aqueous solution of fluorescein sodium salt show clearly fluorescence (Fig. 1c) indicating unambiguously the enclosure of the aqueous solution into the microcapsules.

In order to clarify the mechanism of the formation of well-defined nanocapsules, the polymerization process is monitored by DLS measurements (Fig. 1h). During this process, the particle size and size distribution of the double emulsion decrease, and they reach a plateau (mean diameter 515 nm, PDI 0.14) after 6 h. Since the resulting nanocapsules resemble the structure of the droplets and the creation of monomer swollen micelles as in the case of classical emulsion polymerization is not observed, the polymerization in the present system seems to be confined in the double emulsion droplets and proceeds according to a miniemulsion polymerization mechanism. The significant size decrease can be accounted for by the polymerization-induced shrinkage and for the most part by the conversion of PEOS to silica, which is accompanied by at least 50% weight loss^25^.

### Influence of emulsion composition and pH

**Fig. 2:**
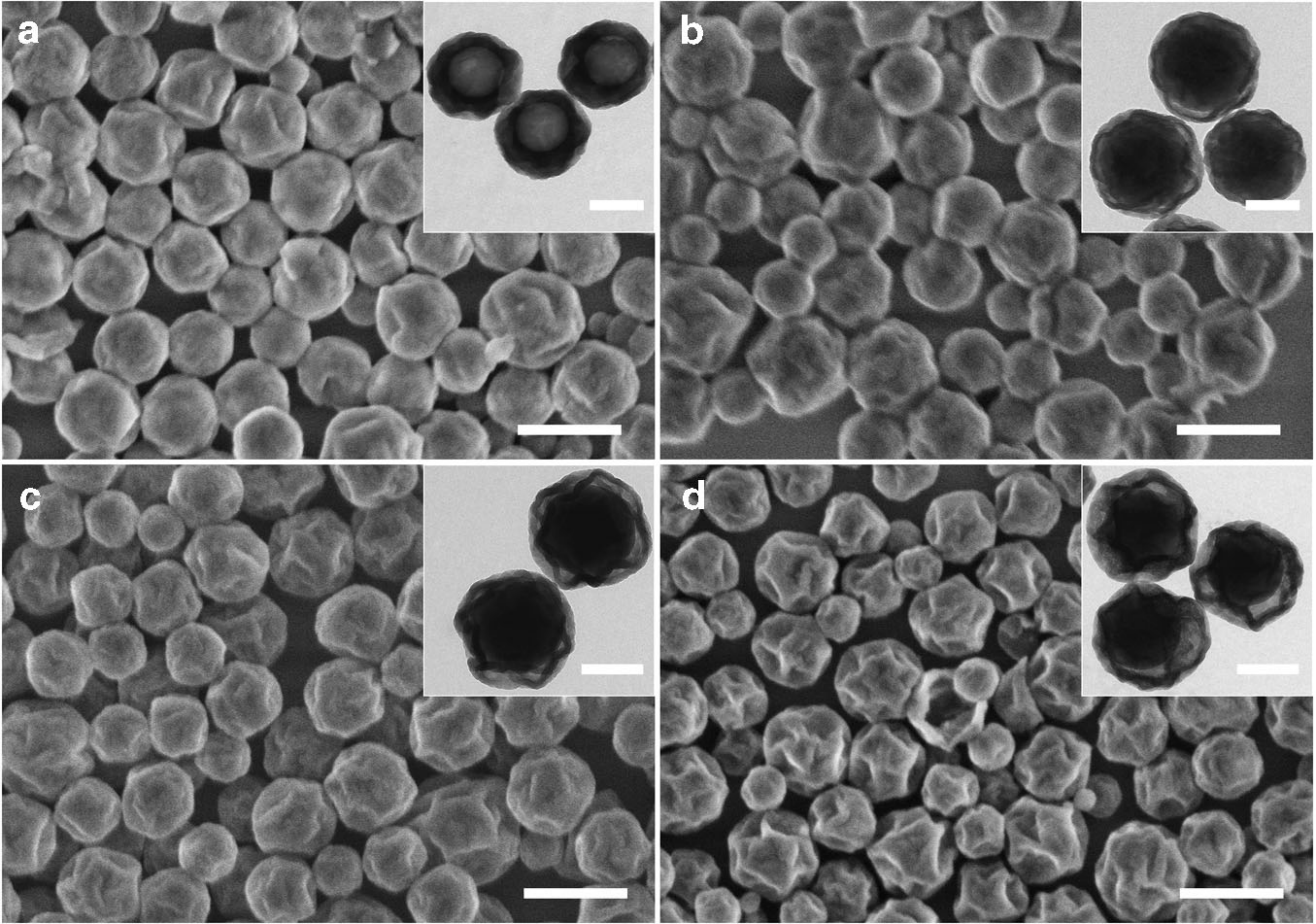
Influence of fractions of inner water phase. The mass ratio of water to the oil phase (PEOS:styrene 1:1) in the primary W/O emulsion is systematically varied: **a** 2:10. **b** 3:10. **c** 4:10. **d** 6:10. The mass ratio of the primary W/O emulsion to the outer water phase is 1:10. FESEM images with scale bars representing 500 nm are shown and the insets are the corresponding TEM images with scale bars of 200 nm

Water@SiO_2_@polystyrene@SiO_2_ nanocapsules described so far are prepared using the primary W/O emulsion where the water-to-oil ratio is 1:10. In principle, the water fraction encapsulated in such nanocapsules can be increased by raising the water-to-oil ratio in the primary W/O emulsion. Here this ratio is systematically varied from 1:10 to 6:10 (entries 2–5 in Table 1). After polymerization of styrene and conversion of PEOS stable milky dispersions are obtained in all cases. According to the electron microscopy data, different water-to-oil ratios result in nanoparticles of different inner structures. By increasing the ratio from 1:10 to 2:10, nanocapsules with a slightly bigger size are formed, and the mean diameter of the inner hollow core increases from 110 to 190 nm (cf. Figs. 1f and 2a). The inner structure of the particles changes dramatically by further increase of the water fraction. A solid core is formed inside a hollow cage, and there is a void between the core and the surrounding shell (Fig. 2b–d). The size of the void increases with the increase of the water fraction as reflected by more wrinkled surface after drying. These so-called nanorattles or yolk-shell nanoparticles find a number of applications in the fields of drug delivery, catalysis, batteries, and so on^27^.

**Table 1:** Recipes for preparation of water-in-oil-in-water double emulsions

Entry no.	Inner water-in-oil (W/O) emulsion				Water-in-oil-in-water (W/O/W) emulsion			
	Styrene	PEOS	Inner water phase		W/O emulsion	Outer water phase		Emulsification method
	(g)	(g)	(g)	pH	(g)	(g)	pH	
1	5	5	1	7.0	3	30	7.0	Ultrasonication
2	5	5	2	7.0	3	30	7.0	Ultrasonication
3	5	5	3	7.0	3	30	7.0	Ultrasonication
4	5	5	4	7.0	3	30	7.0	Ultrasonication
5	5	5	6	7.0	3	30	7.0	Ultrasonication
6	5	5	1	1.0	3	30	7.0	Ultrasonication
7	5	5	1	3.0	3	30	7.0	Ultrasonication
8	5	5	1	5.0	3	30	7.0	Ultrasonication
9	5	5	1	9.5	3	30	7.0	Ultrasonication
10	5	5	1	10.7	3	30	7.0	Ultrasonication
11	5	5	1	7.0	3	30	1.5	Ultrasonication
12	5	5	1	7.0	3	30	2.5	Ultrasonication
13	5	5	1	7.0	3	30	5.0	Ultrasonication
14	5	5	1	7.0	3	30	9.5	Ultrasonication
15	5	5	1	7.0	3	30	10.0	Ultrasonication
16	5	5	1	7.0	3	30	10.7	Ultrasonication
17	5	5	1	7.0	5	50	7.0	Ultra-Turrax 18,000 r.p.m.
18	5	5	1	7.0	5	50	7.0	Ultra-Turrax 12,000 r.p.m.
19	5	5	1	7.0	5	50	7.0	Ultra-Turrax 9000 r.p.m.
20	5	5	1	7.0	5	50	7.0	Ultra-Turrax 4000 r.p.m.

**Fig. 3:**
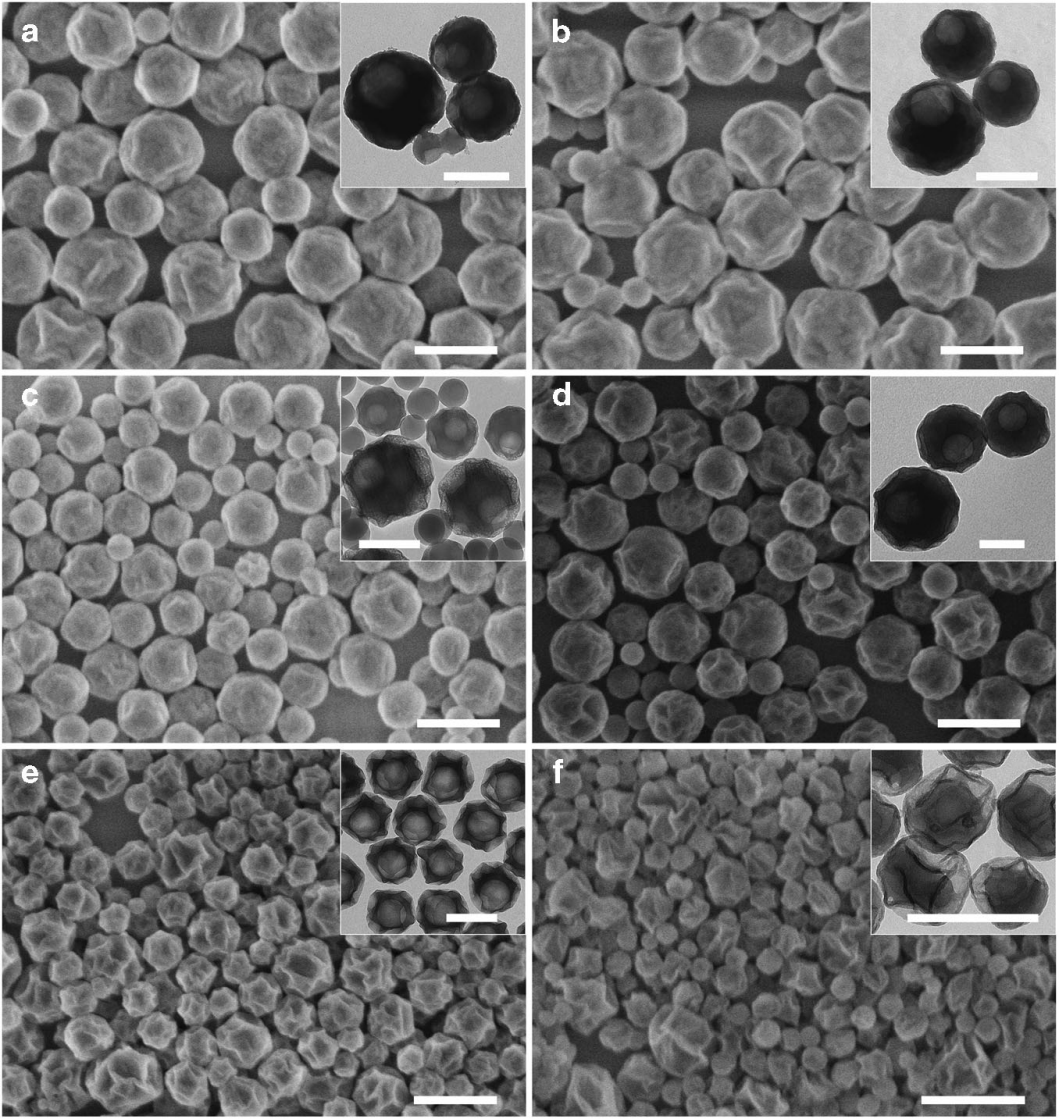
Particles prepared with aqueous phases of different pH. The mass ratio of the inner water phase to the oil phase (PEOS:styrene 1:1) is 1:10, and the primary W/O emulsion to the outer water phase is 1:10. The pH of both inner and outer aqueous phases is delicately adjusted using an ammonia solution or hydrochloric acid. **a** Inner pH 3, outer pH 7. **b** Inner pH 5, outer pH 7. **c** Inner pH 9.5, outer pH 7. **d** Inner pH 7, outer pH 9.5. **e** Inner pH 7, outer pH 10. **f** Inner pH 7, outer pH 10.7. FESEM images with scale bars of 500 nm are shown and the insets are the corresponding TEM images with scale bars of 200 nm

So far all the experiments are performed using deionized water of pH 7 for both inner and outer aqueous phases. As shown in our previous studies, the pH value of the aqueous phase can significantly influence the conversion of PEOS, thus affecting the emulsion stability and morphology of the final capsular structure^24–26, 28^. The pH of both inner and outer aqueous phases is systematically varied from acidic to basic (entries 6–16 in Table 1). When the pH of the outer aqueous phase is kept at 7, the emulsions are generally stable and a capsular structure can be obtained in a broad pH range of the inner aqueous phase (pH 3–10) (Fig. 3a–c). When the inner aqueous phase is too acidic, irregular particle aggregates are formed. Most probably, too fast hydrolysis of PEOS leads to destabilization of inner and possibly also outer interfaces. When the pH of the inner aqueous phase is 10.7, although a stable milky dispersion is formed, solid particles with a core-shell structure are observed. At such high pH, very fast hydrolysis and condensation make PEOS quickly lose its interfacial activity at the inner W/O interface, causing the phase inversion of the inner W/O emulsion. The double miniemulsion is thus transformed to a single O/W miniemulsion whose polymerization leads subsequently to the formation of polystyrene@SiO_2_ core-shell nanoparticles. The pH value of the outer aqueous phase has a more significant impact on this system, and the pH values, at which a well-defined capsular structure is formed, lie in the range of 7–10 (Figs. 1 and 3d, e). In the pH range of 1–5, big solid particles are obtained. Under such acidic conditions, the hydrolysis of PEOS is dominant over condensation, so the interfacial tension between styrene/water decreases continuously with time, indicating an instable O/W interface^25^. Condensation becomes faster over hydrolysis in the pH range of 7–10; therefore, the amphiphilicity of the hydrolyzed PEOS may reach a quasi-stationary state, keeping the O/W interface stable at least during the polymerization reaction. Moreover, due to the fast condensation of PEOS, a solid silica layer is formed quickly at the outer O/W interface for the further stabilization of the droplets. The high pH also endows the particle surface with more charged ionic groups for a highly stable water dispersion. The wrinkling morphology on the capsule surface especially at pH 10 (Fig. 3e) probably is induced by the fast formation of a highly cross-linked silica-like layer from PEOS far before the completion of the polymerization. The volume shrinkage during the polymerization leaves a space between the silica shell and polymer core, which collapses upon drying. An even higher pH (10.7, Fig. 3f) induces the phase inversion of the inner W/O emulsion, and as a result, nanorattles are formed. This is an indication that in these double emulsions addenda and water can migrate through the oil membrane due to the osmotic pressure gradient between the two aqueous phases of different pH^29–31^.

### Conditions for obtaining nanomushrooms

**Fig. 4:**
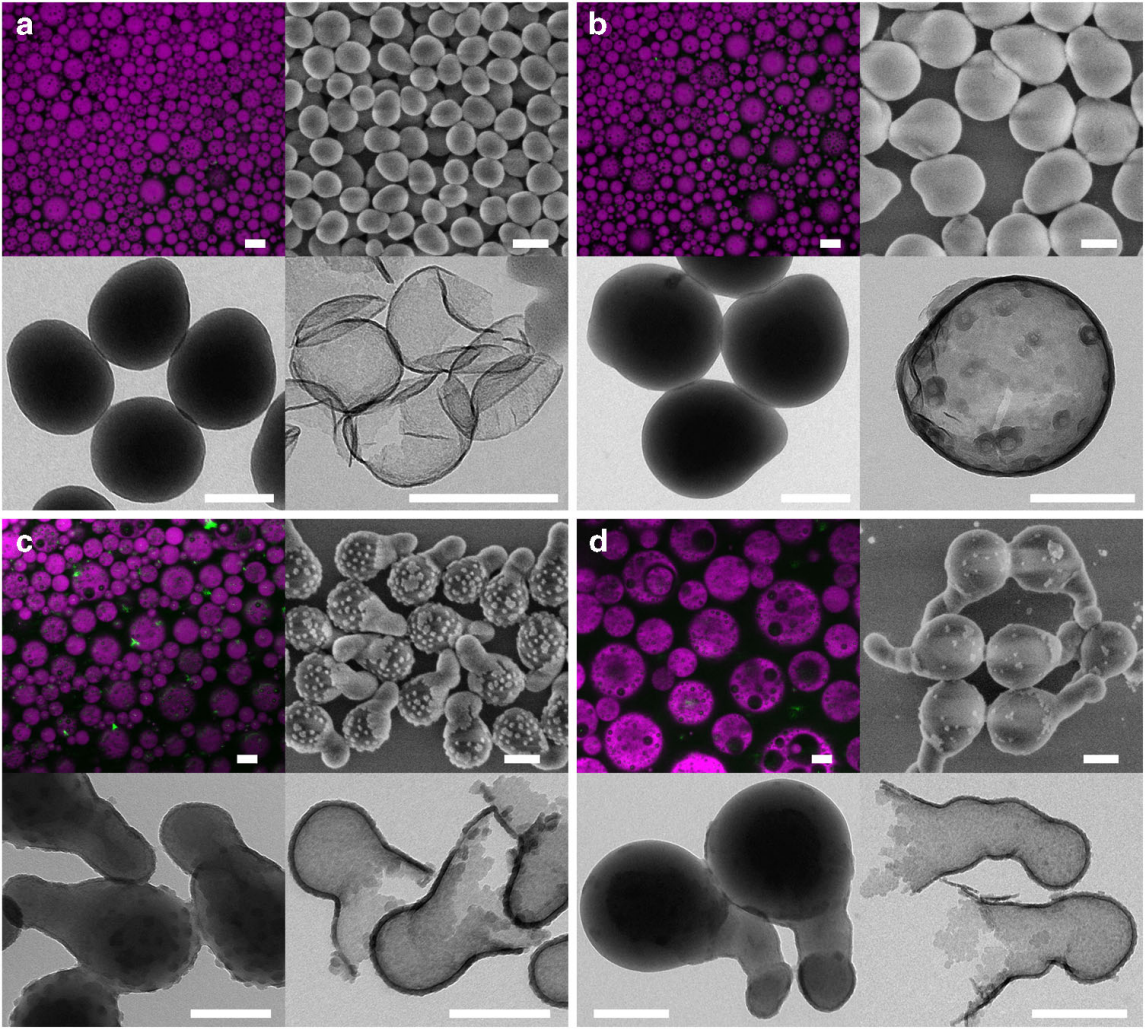
Conversion of microsized multiple emulsions. The mass ratio of PEOS to styrene is 1:1, the inner water phase to the oil phase is 1:10, and the primary W/O emulsion to the outer water phase is 1:10. The pH value of the water phase is 7. Multiple emulsions are prepared using a rotor-stator homogenizer running at different rotation speeds: **a**–**d** 18,000, 12,000, 9000, and 4000 r.p.m. For each speed, a fluorescence micrograph (scale bars = 10 μm) of the emulsion (upper left), a FESEM image (upper right, scale bars = 200 nm), and TEM images of particles before (lower left, scale bars = 200 nm) and after calcination (lower right, scale bars = 200 nm) are shown

In a two-step double emulsion preparation process, dispersion of the primary W/O emulsion into water is generally carried out with a relatively gentle shear force to avoid rupture of the internal water droplets. In the present work, ultrasonication emulsification can be used for both steps, indicating the outstanding stability of the inner W/O emulsion. It can be expected that the use of a gentler dispersion technique in the second emulsification step may result in formation of bigger oil droplets that enclose multiple water droplets. Indeed, multiple emulsions with micrometer sized droplets are formed with a rotor-stator homogenizer, and each oil droplet contains multiple internal water droplets. The size of the oil droplets increases with the decrease of the circumferential speed of the rotor. To our big surprise, by heating the resulting double emulsions under magnetic stirring at 750 r.p.m., the big emulsion droplets fall apart into much smaller nanoparticles (Fig. 4) instead of forming big capsules. At higher rotation speeds of 12,000 and 18,000 r.p.m., solid polystyrene@SiO_2_ core-shell nanoparticles are obtained. These particles have a smooth surface but an oval shape (Fig. 4a, b). By decreasing the rotation speed to 9000 r.p.m., nanoparticles resembling the form of fly agaric mushrooms are observed (Fig. 4c). According to the TEM data, the stipe of the mushroom has a polystyrene@SiO_2_ core-shell structure, and the cap consists of a polystyrene particle decorated with SiO_2_ spots. When the rotation speed is further lowered to 4000 r.p.m., the stipe of the mushroom becomes longer and thinner, and the number of spots on the cap is reduced, particles like ice cream cones are formed (Fig. 4d). In all these cases, no water droplets are observed to be encapsulated inside the nanoparticles.

## Discussion

**Fig. 5:**
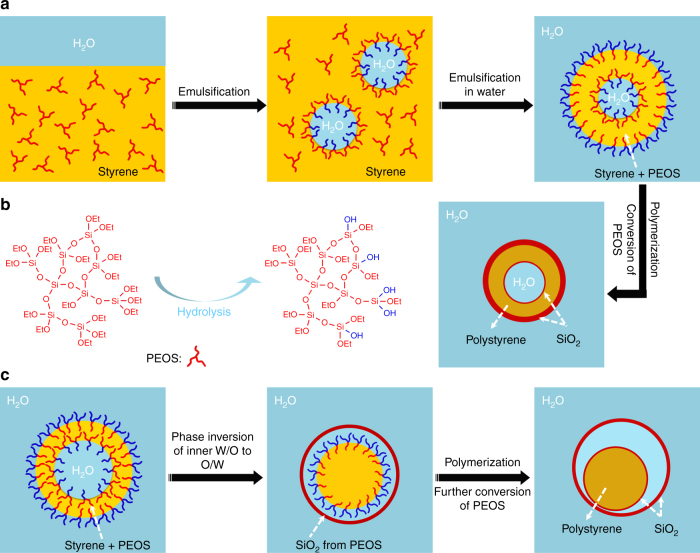
Nanocapsules and nanorattles. **a** A low fraction of internal water phase results in H_2_O@SiO_2_@polystyrene@SiO_2_ nanocapsules and **c** polystyrene@SiO_2_@H_2_O@SiO_2_ nanorattles are obtained with a relatively high fraction of internal water phase. **b** Hydrolysis-induced amphiphilicity of PEOS

The processes for the formation of H_2_O@SiO_2_@polystyrene@SiO_2_ nanocapsules and polystyrene@SiO_2_@H_2_O@SiO_2_ nanorattles are schematically summarized in Fig. 5. In these W/O/W emulsions, PEOS acts as a smart material. It can adjust its degree of hydrolysis to be adapted to both W/O and O/W curved interfaces for their stabilization, thanks to the different rate of hydrolysis at different interfaces. It is well described in our previous work that PEOS is miscible with styrene but not compatible with polystyrene^25^. As the polymerization of styrene proceeds, PEOS macromolecules initially dissolved in styrene are expelled to both interfaces. Due to the faster conversion of PEOS at the outer O/W interface, PEOS macromolecules diffuse preferably toward outside as driven by the osmotic pressure. Thus, a thick silica layer forms at the outer O/W interface, meanwhile a much smaller amount of PEOS reaches the inner W/O interface leading to the formation of a very thin silica layer.

We believe that the formation of a nanorattle structure is induced by phase inversion of the inner W/O emulsion (Fig. 5c). As discussed earlier, the inner W/O emulsion is stabilized by PEOS macromolecules with a low degree of hydrolysis. However, the hydrolysis proceeds further at the W/O interface raising the HLB value of PEOS and eventually PEOS becomes so hydrophilic that it can no more stabilize a W/O emulsion, thus phase inversion takes place. In the case of a low water fraction, the rate of hydrolysis is so slow that the system becomes solidified before the occurrence of the phase inversion. With the increase of the fraction of the inner water phase, the hydrolysis of PEOS is accelerated, so it seems that the inner W/O emulsion is transformed to an O/W emulsion when the oil phase is still liquid. Since the conversion of PEOS at the outer O/W interface is much faster than that at the inner W/O interface, a solid silica layer has already been formed when the phase inversion occurs. Therefore, the phase inversion and polymerization are restricted within the silica cage.

**Fig. 6:**
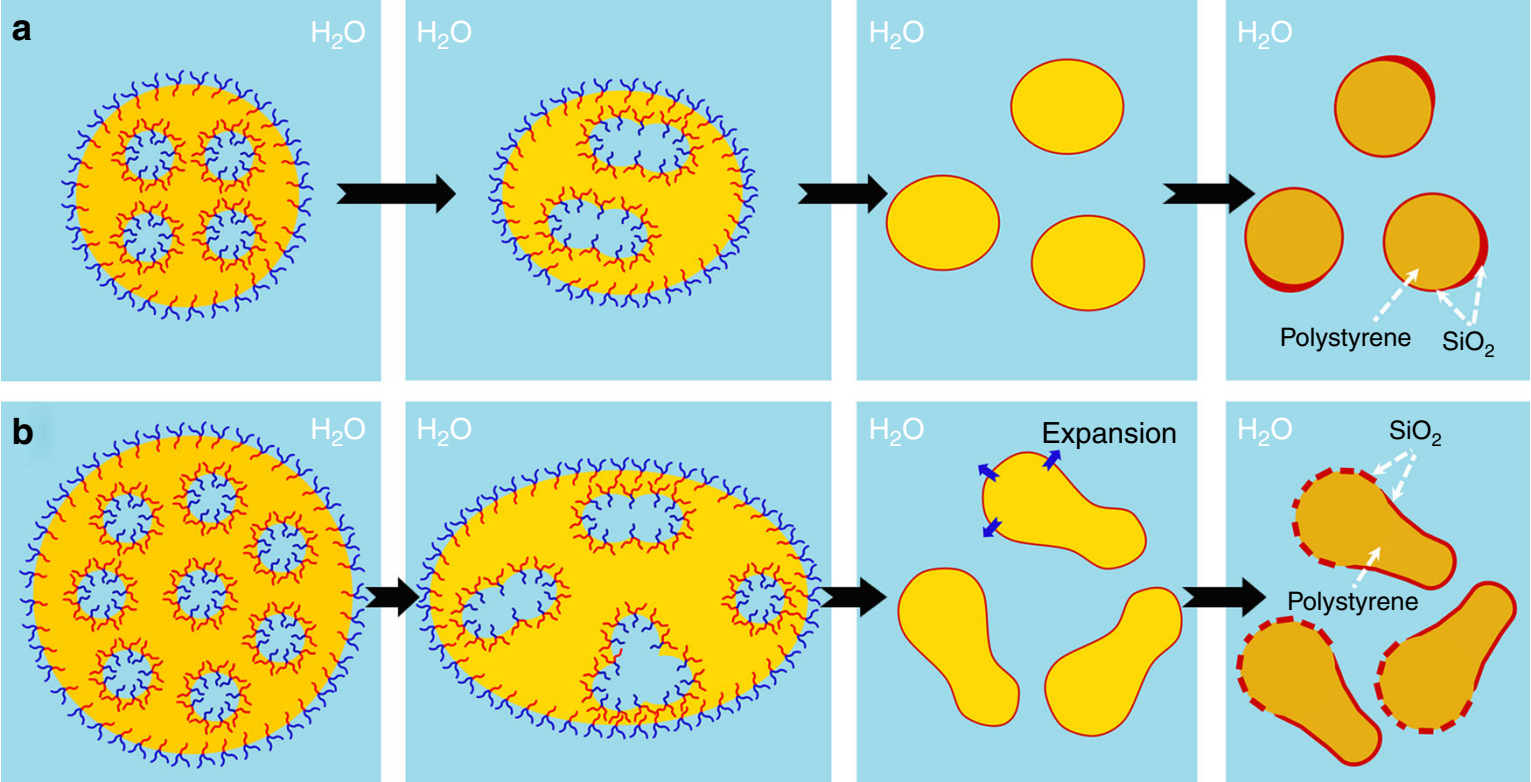
Oval-shaped nanoparticles and Janus-like nanomushrooms. Emulsion droplets containing multiple water droplets in each oil droplet break up to small nanoparticles upon heating. **a** Oval-shaped polystyrene@SiO_2_ particles are formed from relatively small emulsion droplets that cannot be deformed significantly by magnetic stirring. **b** Relatively big emulsion droplets that are largely elongated by magnetic stirring are fragmentized into anisotropic particles, and the recovery of the liquid core to a spherical shape induces the breakage of the already formed silica shell

The fragmentation of big multiple emulsion droplets when a rotor-stator homogenizer is employed in the second emulsification step is most probably the result of interplay between PEOS hydrolysis-induced phase inversion, attraction force between the internal water droplets inside a big oil droplet, and shear force during stirring (Fig. 6). The shear force created by magnetic stirring is too weak to deform largely the relatively smaller emulsion droplets produced by higher shear force; therefore they are broken up by coalescence of the internal water droplets and phase inversion of the inner W/O emulsion, and the nearly spherical shape of the resulting particles is just because of interfacial tension (Fig. 6a). However, the bigger emulsion droplets can be significantly deformed by stirring, leading to the formation of elongated particles (Fig. 6b). A solidified silica-like layer is probably already formed on the surface of these particles, when their core is still made from a viscous solution of polystyrene in styrene. The recovery of the elongated core to a spherical shape causes then the defragmentation of the silica shell, resulting in mushroom-like or ice cream-cone-like nanoparticles. Both types of particles have a Janus-like structure^32^, and the small variance between them can be accounted for by their different deformation degrees. They can stabilize both O/W and W/O emulsions, implying that they exhibit high interfacial activity due to the presence of both hydrophobic and hydrophilic surface properties. These particles have certain similarity to acorn capsules described earlier in the literature, whose formation was attributed to the interplay of three interfacial tensions between pairs of three phases (oil, water, and polymer)^33^. In our case, the mechanism of formation is quite different. The interfacial tension between polystyrene and water is larger than that arising from the polystyrene/PEOS interface, and this is why core-shell particles are formed in the miniemulsion polymerization of the PEOS/styrene system^25^, so the conditions for the formation of acorn capsules cannot be satisfied. Therefore, we believe that the dominating factor is the elasticity of the shell.

Our experiments show that the silica precursor polymer, PEOS, acts as an intelligent material, which is able to stabilize both W/O and O/W interfaces in a W/O/W double emulsion by self-regulating its degree of hydrolysis to different interface curvatures. By using ultrasonication in both emulsification steps, double miniemulsions where each oil droplet encloses only one water droplet are obtained. After polymerization of the oil phase and conversion of PEOS into SiO_2_, the emulsion droplets are solidified to yield H_2_O@SiO_2_@polymer@SiO_2_ nanocapsules. Such a well-defined nanocapsular structure can form in a broad pH range of the inner water phase; meanwhile the pH of the outer water phase should be in the range of 7–10. With increase of the fraction of the inner water phase, phase inversion of the internal W/O emulsion induced by further hydrolysis of PEOS takes places, thus leading to formation of polymer@SiO_2_@H_2_O@SiO_2_ nanorattles. The use of a rotor-stator homogenizer in the second emulsification step results in emulsions with big oil droplets, each of which contains multiple internal water droplets. The emulsion droplets fall apart into small mushroom- or ice cream-cone-like Janus nanoparticles or nearly spherical core-shell nanoparticles after polymerization. It is believed to be the result of interplay between PEOS hydrolysis-induced phase inversion, attraction between internal water droplets inside a big oil droplet, and shear force during stirring. This simple surfactant-free double miniemulsion polymerization technique opens a promising avenue for mass production of various complex hybrid nanostructures that are amenable to numerous applications particularly in the food, cosmetic, pharmaceutical, separation, as well as coating industries.

## Methods

### Materials

Styrene (ReagentPlus^®^, ≥99%) was purchased from Sigma-Aldrich and purified by distillation under vacuum. 2,2′-Azobis(2-methylpropionitrile) (AIBN) was also purchased from Sigma-Aldrich, and recrystallized with ethanol prior to use. Tetraethoxysilane (reagent grade, 98%), acetic anhydride (ACS reagent, ≥98.0%), ammonia solution (ACS reagent, 28–30%), hydrochloric acid (ACS reagent, 37%), fluorescein sodium salt (BioReagent, suitable for fluorescence), and Nile red (BioReagent, ≥98.0%) were purchased from Sigma-Aldrich and tetrakis(trimethylsiloxy)titanium was from ABCR, and they were used as received. Deionized water was used for all experiments.

### Synthesis of PEOS

PEOS was synthesized according to the method published elsewhere^23^. In a 2-L three-neck round-bottom flask equipped with a mechanical stirrer and a 30 cm dephlagmator connected with a distillation bridge tetraethoxysilane (833.32 g), acetic anhydride (408.36 g) and tetrakis(trimethylsiloxy)titanium (4.86 g) were mixed under a nitrogen atmosphere. Under intensive stirring, the mixture was heated to 135 °C using an oil bath. Resulting ethyl acetate was continuously distilled off. Heating was continued until the distillation of ethyl acetate stopped. Afterwards, the product was cooled down to room temperature and dried in vacuum (0.1 mbar) for 2 h. The resulting oily product was treated on a vacuum thin film evaporator (type S 51/31, Normag) equipped with a rotary vane vacuum pump (model RZ-5, Vacuubrand), a magnetic coupling (Buddelberg) for the stirrer (model RZR 2020, Heidolph) operating at the highest level 10, and a heating device. The operating temperature was 150 °C, and the pressure was 0.02 mbar. A yellowish oily liquid (325.47 g) was obtained. The resulting PEOS has the following characteristics: degree of branching 0.54, SiO_2_ content 49.2%, *M*_*n*_ 1740, and *M*_*w*_/*M*_*n*_ 1.9 (measured by gel permeation chromatography in chloroform with evaporative light scattering detector calibrated using polystyrene standards).

### Preparation of water-in-oil-in-water double emulsions

Styrene, AIBN, and PEOS were firstly mixed to form a homogeneous oil phase. The W/O/W double emulsion was prepared using a two-step emulsification method. A primary water-in-oil (W/O) emulsion was first prepared by dispersing water into the oil phase under ultrasonic irradiation for 5 min (Branson Sonifier 450 cell disrupter, 3 mm microtip, 0.9 time circle, 247 W output). The resulting W/O emulsion was then dispersed into an aqueous phase to obtain W/O/W double emulsion with the same ultrasonic treatment for 5 min or a rotor-stator homogenizer (T 18 digital ULTRA TURRAX^®^, IKA) for 10 min. The detailed experimental recipes are listed in Table 1.

### Synthesis of polystyrene/silica hybrid nanostructures

The milky W/O/W double emulsion was injected into a nitrogen-filled three-neck flask in an oil bath. Under magnetic stirring running at 750 r.p.m., the temperature of the oil bath was raised to 70 ^o^C to trigger the polymerization, and the heating lasted for 24 h. Afterwards, the reaction mixture was cooled down to room temperature. The resulting latex particles were isolated by centrifugation and re-dispersed in water or freeze-dried for further characterization. Pure silica materials were obtained by calcination in a muffle oven at 600 °C in air for 5 h.

### Confocal laser scanning microscopy

Confocal laser scanning microscopy was performed on a Leica TCS SP8 to visualize the mixture of aqueous phase fluorescently labeled with fluorescein sodium salt and oil phase labeled with Nile red. The emulsions or aqueous dispersions of resulting nanocapsules were placed on a glass substrate and covered with a coverslip. The samples were imaged using different excitation laser lines and separate detectors, corresponding to the fluorescence profiles of the used fluorescent dyes, respectively.

### Dynamic light scattering measurements

DLS measurements of particles in water were performed on a Malvern Zetasizer Nano Series at a scattering angle of 173° at 25 °C. Before measurements, the stock dispersions were diluted to a particle concentration of 1.5 wt.‰.

### Field-emission scanning electron microscopy

Field-emission scanning electron microscopy measurements were performed on a Hitachi S4800 high-resolution field-emission scanning electron microscope with an accelerating voltage of 1.5 kV. A droplet of sample dispersion was placed on a silicon wafer substrate and air-dried under ambient conditions.

### Transmission electron microscopy

TEM measurements were carried out on a Zeiss Libra 120 TEM. The accelerating voltage was set at 120 kV. The samples were prepared by placing a droplet of the diluted sample dispersion on a Formvar-carbon-coated copper grid with 200 mesh.

### Fourier-transform infrared spectroscopy

FT-IR spectra were recorded on a Nicolet 60 SXR FT-IR spectrometer using KBr pellet technique. The samples were freeze-dried before measurements.

### Data availability

The authors declare that the data supporting the findings of this study are available within the article. All other relevant source data are available from the corresponding author upon reasonable request.

## Electronic supplementary material

Peer Review File
