# Supplementary material for: Hybrid nanostructured particles via surfactant-free double miniemulsion polymerization
Source: Nat Commun. 2018 May 15;9:1918. doi: 10.1038/s41467-018-04320-7 (PMC5953918; doi:10.1038/s41467-018-04320-7)
Supplement: Supplementary file 1 — Peer Review File [file 41467_2018_4320_MOESM1_ESM.pdf]

Reviewers' comments:

Reviewer #1 (Remarks to the Author):

The authors of this paper report on a versatile technology platform for synthesis of various hybrid nanostructured particles including nanocapsules, nanorattles and “nanomushrooms”. In their work, the main idea is based on a simple silica precursor polymer, hyperbranched polyethoxysiloxane (PEOS), which is interfacial active due to hydrolysis at the oil/water interface. According to the manuscript, in contrast to classical emulsifiers, PEOS is reactive and its properties keep changing during the reaction, so the system containing PEOS can easily be manipulated by varying the preparation conditions to result in different fascinating nanostructures. The described in the draft process seems indeed simple and scalable, so I believe that it should be of a considerable interest to materials and nanotechnology communities. The manuscript is well organized and clearly written. The proposed mechanisms of structure formation are feasible, and the conclusions are well supported by the experimental data. Therefore, I would like to recommend publishing this work in Nature Communications after minor revision and addressing some aspects mentioned below.

1) The process description (first paragraph of the “Results” section) is somewhat not clear. Should not the resulting emulsion after catastrophic phase inversion be O/W? Why then in the next sentence the resulting emulsion is called W/O? The authors report gelation of the initial W/O emulsion with time. Is this gelled phase emulsified in the next step? If the second step would be applied before the gelation takes place, what would be the morphology of the emulsion?

2) The core-shell morphologies referred by the authors as mushroom- or ice-cream-cone-like probably have the same origin as earlier described acorn capsules (Loxley, Vincent J. Colloid Interface Sci. 1998, 208, 49). The mechanism of formation in that work was attributed to the interplay of three interfacial tensions between pairs of the three phases (oil, water, and polymer – PEOS in the case of the present work). If the shell–core surface energy is large, then separation between phases, i.e. the formation of acorn capsules is observed. I suggest that the authors compare their system to this earlier classical knowledge. What is the dominating factor – the surface tension (which can be triggered by pH) or the elasticity of the shell?

Reviewer #2 (Remarks to the Author):

Y. Zhao et al. "Hybrid Nanostructures Templated by Surfactant-Free Double Miniemulsions – From Nanocapsules and Nanorattles to Janus-like Nanomushrooms"

This manuscript describes an elegant and seemingly simple way to stabilise polymerisable double (or multiple) w/o/w miniemulsions using a hyperbranched polyethoxysiloxane, which is oil soluble and hydrolyses only at the o/w interface. This leads to very stable multiple emulsions. The hydrolysis causes the polyethoxysiloxane to form a silica layer. The polymerisation of the emulsions results in the formation of "nanocontainers". Control of the composition of the aqueous phase, i.e. pH, allows to control the hydrolysis reaction of the polyethoxysiloxane and so of the final structure of the polymerised nanocontainers. The evidence is really convincing and the manuscript is well-written.

However, I still have questions; the authors claim the produced polymer/silica nanocomposite capsules can be loaded with an aqueous "payload" (or an water-soluble active ingredient) but fall to provide proof - especially since the water seems to evaporate from the capsules.

Furthermore; the authors claim that the "nano rattles" have a solid core and a spongy outer structure and point to TEM images but I cannot see this. The TEM images do not seem to suggest a spongy structure - maybe its the resolution or?

Also, the authors claim they produced Janus structures with an ice-cone like appearance, which they may well be but Janus particles should have not only a different surface structure but also different surface properties - and also here the proof is derived only from TEM images. If the silica is indeed concentrated on one size of the particles and the other consists of PS, they should also preferentially adsorb at o/w interfaces.

And finally: do you really provide a route to "mass production"? The polyethoxysiloxane, as I understand it, was synthesised by you.

Nevertheless, this manuscript reports a neat way to the synthesis of nanocomposite containers; PS reinforced with a silica layer. The organic component can be removed and one obtains silicanano shells. Moreover, the structure can of this nanocomposite particles can be contained by controlling the emulsification conditions and/or the aqueous phase composition. After, revision this manuscript should be published.

Reviewer #3 (Remarks to the Author):

This is truly an outstanding paper. I appreciate not only the very interesting results, but especially the very convincing interpretations. The test is well written and comprehensible; the two schemes are very helpful.

Although it is an extension of previous work, I consider it as sufficiently innovative and relevant to be published in Nature Communications.

Typos: page 17: ice-cream not scream

I only have one question: in patent EP 3124112 A1 (WO2017016636A1), a similar, if not the same strategy is described, and also water-in-oil-in-water double emulsions are mentioned with droplet sizes in the nanometre regime. What is the difference between the present manuscript and the content of this patent?

NCOMMS-17-32306, Point-by-point response

Reviewer #1 (Remarks to the Author):

The authors of this paper report on a versatile technology platform for synthesis of various hybrid nanostructured particles including nanocapsules, nanorattles and “nanomushrooms”. In their work, the main idea is based on a simple silica precursor polymer, hyperbranched polyethoxysiloxane (PEOS), which is interfacial active due to hydrolysis at the oil/water interface. According to the manuscript, in contrast to classical emulsifiers, PEOS is reactive and its properties keep changing during the reaction, so the system containing PEOS can easily be manipulated by varying the preparation conditions to result in different fascinating nanostructures. The described in the draft process seems indeed simple and scalable, so I believe that it should be of a considerable interest to materials and nanotechnology communities. The manuscript is well organized and clearly written. The proposed mechanisms of structure formation are feasible, and the conclusions are well supported by the experimental data. Therefore, I would like to recommend publishing this work in Nature Communications after minor revision and addressing some aspects mentioned below.

1) The process description (first paragraph of the “Results” section) is somewhat not clear. Should not the resulting emulsion after catastrophic phase inversion be O/W? Why then in the next sentence the resulting emulsion is called W/O? The authors report gelation of the initial W/O emulsion with time. Is this gelled phase emulsified in the next step? If the second step would be applied before the gelation takes place, what would be the morphology of the emulsion?

We agree that our description is not clear enough. We stated that “the catastrophic phase inversion from W/O to O/W type takes place when the water fraction is raised above 67%”, and this sentence can cause confusion. Therefore, we have replaced the word “when” by “**if**” and added “**At lower water fraction**” at the beginning of the following sentence. Actually, the second emulsification is carried out before gelation takes place, so “**before gelation**” has been added on page 6 for the clarity.

2) The core-shell morphologies referred by the authors as mushroom- or ice-cream-cone-like probably have the same origin as earlier described acorn capsules (Loxley, Vincent J. Colloid Interface Sci. 1998, 208, 49). The mechanism of formation in that work was attributed to the interplay of three interfacial tensions between pairs of the three phases (oil, water, and polymer – PEOS in the case of the present work). If the shell–core surface energy is large, then separation between phases, i.e. the formation of acorn capsules is observed. I suggest that the authors compare their system to this earlier classical knowledge. What is the dominating factor – the surface tension (which can be triggered by pH) or the elasticity of the shell?

We thank the reviewer for drawing our attention to this important paper. It is now cited on page 20 as reference 33. Although the mushroom- or ice-cream-cone-like particles look similar to the acorn capsules described in this paper, the mechanism of formation is quite different. The interfacial tension between polystyrene and water is larger than that arising from the polystyrene/PEOS interface, and this is why core-shell particles are formed in the miniemulsion polymerization of the PEOS/styrene system, so the conditions for the formation of acorn capsules cannot be satisfied. Therefore, we believe that the dominating factor in our case is indeed the elasticity of the shell as the reviewer mentioned. The following text has been added on page 19-20.

These particles have certain similarity to acorn capsules described earlier in the literature, whose formation was attributed to the interplay of three interfacial tensions between pairs of three phases (oil, water and polymer).[33] In our case the mechanism of formation is quite different. The interfacial tension between polystyrene and water is larger than that arising from the polystyrene/PEOS interface, and this is why core-shell particles are formed in the miniemulsion polymerization of the PEOS/styrene system,[25] so the conditions for the formation of acorn capsules cannot be satisfied. Therefore, we believe that the dominating factor is the elasticity of the shell.

Reviewer #2 (Remarks to the Author):

This manuscript describes an elegant and seemingly simple way to stabilise polymerisable double (or multiple) w/o/w miniemulsions using a hyperbranched polyethoxysiloxane, which is oil soluble and hydrolyses only at the o/w interface. This leads to very stable multiple emulsions. The hydrolysis causes the polyethoxysiloxane to form a silica layer. The polymerisation of the emulsions results in the formation of "nanocontainers". Control of the composition of the aqueous phase, i.e. pH, allows to control the hydrolysis reaction of the polyethoxysiloxane and so of the final structure of the polymerised nanocontainers. The evidence is really convincing and the manuscript is well-written.

However, I still have questions; the authors claim the produced polymer/silica nanocomposite capsules can be loaded with an aqueous "payload" (or an water-soluble active ingredient) but fall to provide proof - especially since the water seems to evaporate from the capsules.

We have carried out a fluorescence optical microscopy study on the resulting polymer/silica nanocomposite capsules containing an aqueous solution of fluorescein sodium salt. The obtained fluorescence micrograph is shown as **Fig. 1c** to confirm the loading with an aqueous "payload". The following text has been added on page 9.

The resulting microcapsules prepared with an internal aqueous solution of fluorescein sodium salt show clearly fluorescence (Fig. 1c) indicating unambiguously the enclosure of the aqueous solution in the microcapsules.

Furthermore; the authors claim that the "nano rattles" have a solid core and a spongy outer structure and point to TEM images but I cannot see this. The TEM images do not seem to suggest a spongy structure - maybe its the resolution or?

On page 11 we claimed that "a solid core is formed inside a hollow cage, and there is a void between the encapsulated cargo and the surrounding shell" and there is no spongy outer structure mentioned. We think it might not be appropriate to use the term "encapsulated cargo", so this sentence has been modified as follows.

A solid core is formed inside a hollow cage, and there is a void between the core and the surrounding shell.

The schema of a "nanorattle" is shown in Scheme 1c.

Also, the authors claim they produced Janus structures with an ice-cone like appearance, which they may well be but Janus particles should have not only a different surface structure but also different surface properties - and also here the proof is derived only from TEM images. If the silica is indeed concentrated on one size of the particles and the other consists of PS, they should also preferentially adsorb at o/w interfaces.

We agree with the reviewer that Janus particles are particles whose surfaces have two or more distinct physical properties and therefore they exhibit high interfacial activity. We tested the mushroom like and ice-cone like particles in the preparation of O/W and W/O emulsions, and found that these particles can stabilize both types of emulsions, implying that have high interfacial activity due to the presence of both hydrophobic and hydrophilic surface properties. The following text has been added on page 19.

They can stabilize both O/W and W/O emulsions, implying that they exhibit high interfacial activity due to the presence of both hydrophobic and hydrophilic surface properties.

And finally: do you really provide a route to "mass production"? The polyethoxysiloxane, as I understand it, was synthesised by you.

Currently we are working together with a company to use polyethoxysiloxane (PEOS) for microencapsulation applications. It has been shown by the company that the PEOS synthesis can be easily scaled up, and they have already produced 200 kg for their internal use.

Nevertheless, this manuscript reports a neat way to the synthesis of nanocomposite containers; PS reinforced with a silica layer. The organic component can be removed and one obtains silica nano shells. Moreover, the structure can of this nanocomposite particles can be contained by controlling the emulsification conditions and/or the aqueous phase composition. After, revision this manuscript should be published.

Reviewer #3 (Remarks to the Author):
This is truly an outstanding paper. I appreciate not only the very interesting results, but especially the very convincing interpretations. The test is well written and comprehensible; the two schemes are very helpful.

Although it is an extension of previous work, I consider it as sufficiently innovative and relevant to be published in Nature Communications.

Typos: page 17: ice-cream not scream.

It has been corrected.

I only have one question: in patent EP 3124112 A1 (WO2017016636A1), a similar, if not the same strategy is described, and also water-in-oil-in-water double emulsions are mentioned with droplet sizes in the nanometre regime. What is the difference between the present manuscript and the content of this patent?

WO2017016636A1 is a patent submitted by part of the authors (Yongliang Zhao, Martin Möller and Xiaomin Zhu), in which the double emulsion technique is described. This patent has been mentioned by us in the section “Competing financial interests” stating that “The results presented in this paper form a part of a patent submitted by Y.Z., X.Z., and M.M.”. The patent is focused on microencapsulation of substances by using polyethoxysiloxane (PEOS), and it includes not only the double emulsion technique, but also PEOS-assisted single emulsion strategies. The present manuscript goes far beyond the patented double emulsion technology. It reports on an in-depth investigation on the PEOS-stabilized double emulsion systems by varying systematically reaction conditions and providing comprehensive mechanisms to explain the formation of different hybrid nanostructured particles.

REVIEWERS' COMMENTS:

Reviewer #1 (Remarks to the Author):

After revision, I recommend this manuscript for publication.

Reviewer #2 (Remarks to the Author):

I read the manuscript "Hybrid Nanostructured Particles via Surfactant-Free Double Miniemulsion Polymerization" by Y. Zhao et al. again. As already stated in my original review this manuscript deserves to be published in Nature Comm. The work is exciting and the discussion of the results sound. I also read the comments raised by the other reviewers all our comments were fully addressed and additional results in support of the claims made in the original version are now provided and substantiated. I do recommend this manuscript to be published.

Reviewer #3 (Remarks to the Author):

The authors satisfactorily answered to the raised comments and concerns of the reviewers. Therefore, I recommend now publication of the revised manuscript as it stands.

NCOMMS-17-32306A, Point-by-point response

Reviewer #1 (Remarks to the Author):

After revision, I recommend this manuscript for publication.

Reviewer #2 (Remarks to the Author):

I read the manuscript "Hybrid Nanostructured Particles via Surfactant-Free Double Miniemulsion Polymerization" by Y. Zhao et al. again. As already stated in my original review this manuscript deserves to be published in Nature Comm. The work is exciting and the discussion of the results sound. I also read the comments raised by the other reviewers all our comments were fully addressed and additional results in support of the claims made in the original version are now provided and substantiated. I do recommend this manuscript to be published.

Reviewer #3 (Remarks to the Author):

The authors satisfactorily answered to the raised comments and concerns of the reviewers. Therefore, I recommend now publication of the revised manuscript as it stands.

We thank sincerely the reviewers for their positive opinion on the revised version of our paper.
